# Multiscale analysis of hydrogen-induced softening in f.c.c. nickel single crystals oriented for multiple-slips: elastic screening effect

**DOI:** 10.1038/s41598-019-49420-6

**Published:** 2019-09-10

**Authors:** I. M. A. Ghermaoui, A. Oudriss, A. Metsue, R. Milet, K. Madani, X. Feaugas

**Affiliations:** 1 LMPM, Université de Djilali Liabes, Faculté de Technologie, BP 89 Cité Ben M’Hidi, 22000 Sidi Bel Abbes, Algeria; 2 La Rochelle University, LaSIE UMR CNRS 7356, Av. Michel Crépeau, 17042 La Rochelle, Cedex 1 France

**Keywords:** Mechanical properties, Applied physics

## Abstract

Hydrogen-deformation interactions and their role in plasticity are well accepted as key features in understanding hydrogen embrittlement. In order to understand the nature of the hydrogen-induced softening process in f.c.c. metals, a substantial effort was made in this study to determine the effect of hydrogen on the tensile stress-strain behavior of nickel single crystal oriented for multiple-slips. It was clearly established that the hydrogen softening process was the result of a shielding of the elastic interactions at different scales. Hydrogen-induced softening was then formalized by a screening factor S of 0.8 ± 0.05 for 7 wppm of hydrogen, which can be incorporated into standard dislocation theory processes. The amplitude of softening suggests that the shielding process is mainly responsible for the stress softening through the formation of vacancy clusters, rather than a direct impact of hydrogen. This effect is expected to be of major importance when revisiting the impact of hydrogen on the processes causing damage to the structural alloys used in engineering.

## Introduction

The effect of hydrogen on the mechanical behavior of metallic materials has been widely studied^1–5^. In a large majority of these studies hydrogen had a detrimental effect, causing materials to fail at loads well below those that induce failure in the absence of hydrogen. This effect is known as “Hydrogen Embrittlement” and essentially describes a significant reduction in macroscopic ductility and ultimate tensile strength, as well as a change in the fracture mode.

Hydrogen-related failures are attributed to several mechanisms, but the main contribution seems to be the result of hydrogen/plasticity interactions as hydrogen enhances localized plasticity (HELP). Many studies support a role for the shielding effect in fcc metals^2,6–13^. Depending on solute content, solute diffusivity and strain rate, softening and hardening processes were observed^1,8,9,13–21^ and formalized in a text book^2^.

A shielding effect is observed when hydrogen mobility is greater than dislocation mobility, leading to a reduction in elastic interaction between dislocations and consequently promoting stress softening. In contrast, when hydrogen mobility is similar to dislocation mobility, the solute pinning process can occur and promote stress hardening due to the solute drag mechanism^2,13^. A direct link between the fundamental properties of dislocation affected by hydrogen and macroscopic behavior is still subjective. Indeed, the influence of the solute on the collective movement and distribution of storage dislocations is a complex subject that would require more in-depth analysis^22,23^.

Some studies showed an impact of hydrogen on the localization of strains observed on the surface as a form of deformation bands^24,25^. The link between this localization and the deformation structures developed during tensile loading has only recently been studied in single-slips and the impact on hardening clearly demonstrated^22^. In this orientation, the formation of geometric necessary boundaries (GNBs, defined as a type I pattern^26^) both screen the long-range internal stress fields and decrease the mean free path of mobile dislocations. As a consequence, the formation of GNBs concentrates deformations in specific slip bands. It has been established that hydrogen decreases GNB spacing through a shielding effect which promotes the multiplication of dislocation sources at the early stage of deformation. The decrease in GNB spacing directly impacts strain hardening by decreasing the length scale^22^.

In contrast, the impact of hydrogen on strain hardening and the dislocation pattern under multiple-slips is still controversial^22^. In these conditions, without hydrogen, an extensive investigation of dislocation distribution in hardening stages II and III illustrated the impact of grain orientation on the morphology of dislocations cells^26,27^. For a tensile axis near the textured fiber (110)-(111), a type III microstructure dislocation pattern is formed with similar morphology to that observed in a type I microstructure with GNBs. In contrast to the type I pattern, this microstructure is subdivided by ‘non-crystallographic’ boundaries, in accordance with a deviation of the boundary plane from primary, conjugate and cross-slip planes^28^. In contrast to type I and III patterns, grains in a polycrystal or single-crystal with a tensile axis in the vicinity of the <001> crystallographic orientation do not form extended planar boundaries but a well-defined equiaxed cell with mainly IDBs (Incidental Dislocations Boundaries) as dislocation walls due to a high degree of multiple-slips. For these orientations, the effect of a solid solution of hydrogen on dislocation organization is unclear. In early work on the subject, no significant modification was reported in strained polycrystalline microstructures^29–31^. More recently, Girardin *et al*. reported a decrease in equiaxed dislocation cell size in nickel in single and multiple-slip oriented single-crystals^22^. This result was confirmed by Wang *et al*.^23^ for polycrystalline nickel samples subjected to high-pressure torsion (HPT), but was not clearly established by Harris *et al*.^32^ for polycrystalline nickel samples subjected to tensile straining of 10% at 77 K and 300 K. According to Girardin *et al*., hydrogen seems to have less impact on equiaxed dislocation cell size than on GNB spacing (3 times lower)^22^. The consequences of these observations on hardening rate have not been clearly established and are still under debate.

In this study, a substantial effort was made to investigate the effect of hydrogen in solution on the tensile stress-strain behavior of nickel oriented for multiple-slips (001) using a multi-scale approach. The underlying mechanisms for hydrogen-induced softening are discussed in relation to systematic experiments and analyses of hardening rate, internal stress, apparent activation volume and the organization of dislocations.

## Results

### Mechanical tests

**Figure 1:**
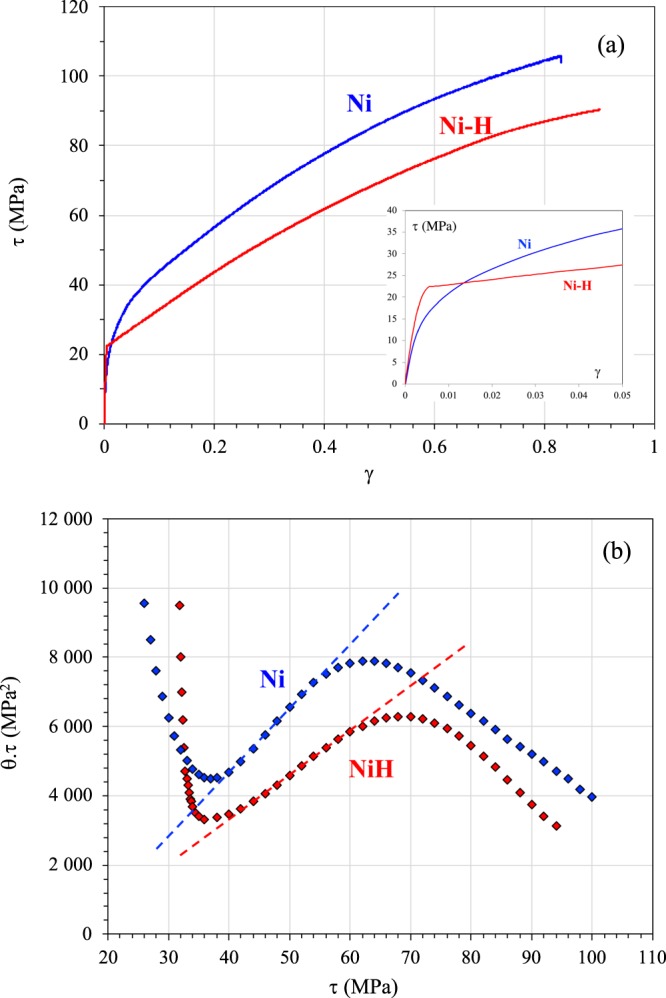
Stress-strain curve (**a**) and hardening rate as a function of shear stress (**b**) for Ni and Ni-H. The zoom on (**b**) highlights the hardening process, which occurs at the early stage of deformation. The slopes on picture 1b correspond to the hardening rate in stage II (θ_II_) and can be used to define the domain of stages I, II and III.

The mechanical behavior of Ni and Ni-H (001) single-crystals at 300 K was assessed based on a standard shear stress-strain curve (τ *versus* γ) and changes in work-hardening (θ) (Fig. 1). The latter is generally defined using a Kocks-Mecking diagram (Fig. 1b) where the τ.θ *vs* τ curve can be used to define the different hardening stages^33,34^. The linear behavior observed in stage II offers the opportunity to define two other stages: a pseudo-stage I and a stage III before and after stage II. With the addition of hydrogen, the early stages of plastic strain showed an increase in flow stress and an extended pseudo-stage I in terms of deformation. This behavior has previously been reported by Girardin *et al*. for nickel single-crystal oriented for single-slip^8,9,22^. The increase in the initial magnitude of flow stress (1.5 MPa for nickel at 300 K according to Windle *et al*. 1968^18^) near the elasto-plastic transition was around 20 MPa with the addition of hydrogen (Fig. 1a). This value corresponds to the maximum viscous drag stress of a solute atmosphere calculated from static strain ageing experiments used to measure the saturated dislocation pinning force as a function of hydrogen content^8,9,35^.

The partition of the stress-strain curve into hardening stages was initially based on studies of a deformed single crystal single slip-oriented. The extension of these notions to multiple slip orientation single crystals and polycrystals is a subject that has led to many debates in the past. The transition between stage I and stage II is thought to be connected to the strong activity of a second slip system which crosses the first. At the early stage of plasticity (pseudo stage I), for orientation in the vicinity of the [001] orientation, only one set of slip lines was observed on the surface in accordance with the occurrence of two collinear slip systems^36,37^. Based on these considerations, pseudo stage I is defined as a transition between the onset of plasticity (yield stress) and stage II, with a predominance of only one slip system at the early stage of deformation, despite the multi-slip orientation. The delay in the emergence of stage II with the addition of hydrogen is generally associated with the fact that hydrogen favors planar glide due to a decrease in the probability of cross-slip, and prolongs the “pseudo single slip regime”. This effect can be attributed to a shielding of the pair interactions between partial dislocations induced by hydrogen solute^2^.

In stages II and III, stress softening was observed with the addition of hydrogen (Fig. 1a). This was expressed as a decrease in hardening rate θ_II_ in stage II (194 MPa for Ni and 124 MPa for Ni-H) and a delay in the transition between stages II and III (stress/strain 70 MPa/0.51 for Ni-H and 63 MPa/0.26 for Ni). Stage III is generally associated with a recovery mechanism in relation to cross-slip activity. According to Escaig’s model^38^, it was related that τ_III_ increases as function of the stacking fault width. As a consequence, the fact that τ_III_ was higher for Ni-H than Ni was due to the effect of hydrogen on stacking fault energy, and/or on the dissociation width of screw dislocation and the ease of planar slip, which delayed the occurrence of cross-slip^2^.

### Thermal activation processes

**Figure 2:**
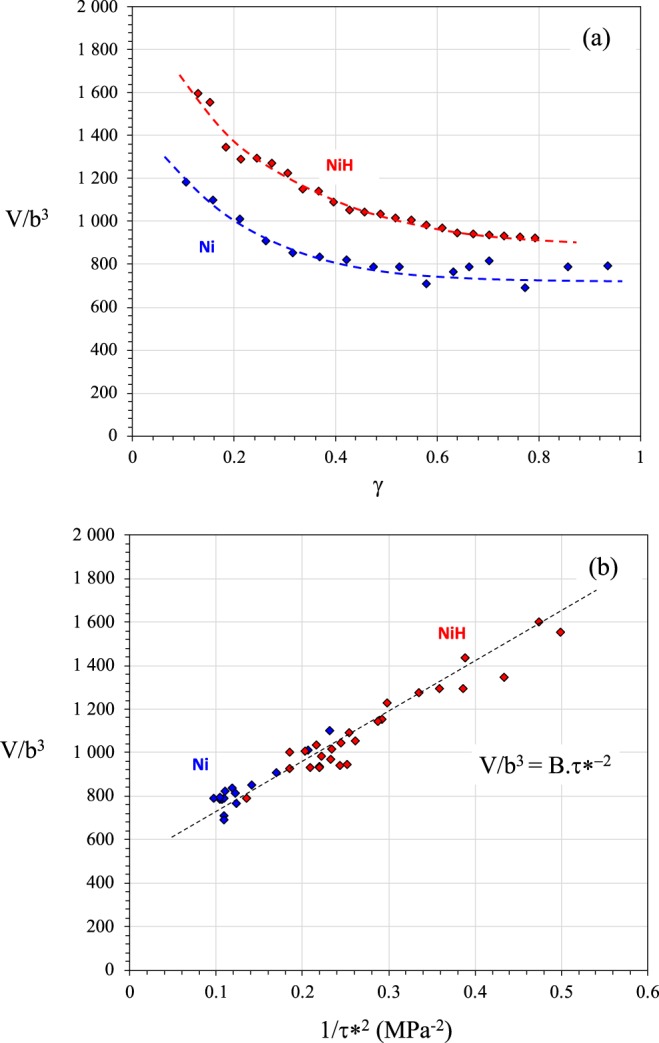
Normalized activation volume V/b^3^ as a function of shear strain for Ni and Ni-H (a). Normalized activation volume as a function of the ratio 1/τ*^2^ where τ* is the thermal component of short-range internal stress.

The isothermal stress relaxation technique can be used to determine the activation parameters for dislocation slip in nickel and the ways in which hydrogen can affect this. The normalized activation volume V/b^3^ as a function of stress is shown in Fig. 2. Adding hydrogen increased the activation volume in the strain range studied (Fig. 2a). Given the thermally activated mechanisms involved in crystal plasticity, the change in V/b^3^ *versus* (1/τ*)^n^ could be used to identify the physical mechanism associated with dislocation motion (n is a coefficient and τ* is thermal part of the stress). A linear relation was obtained for n = 2, which suggests that for pure Ni and Ni-H the dominant barriers were probably forest dislocations (Fig. 2b). Additionally, the slope of V/b^3^ *versus* (1/τ*)^2^ doesn’t depend on hydrogen content and was 2147 MPa^2^. In accordance with a pinning model, the forest-hardening process^39,40^ can be expressed as V/b^3^ = (π/8) [Mαµk_0_]^2^(τ*)^−2^ with α the elastic interaction coefficient, µ the shear modulus, M the Taylor factor (M = 3) and k_0_ a constant relative to the pinning distance (or mean free path) expressed as k_0_/$\sqrt{\rho }$ in the specific case of a forest dislocation distribution (ρ is the dislocation density). Consequently, the B = (π/8) [Mαµk_0_]^2^ slope remained constant with the addition of hydrogen. At the early stage of deformation, the strain rate was very low and so the energy of the obstacle could be deduced as V × τ*. The energy associated with the glide across the forest dislocation was 0.43 eV and 0.23 eV for Ni and Ni-H, respectively, which highlights a softening effect in terms of the energy barrier for dislocation mobility. Combette, Sirois and Birnbaum reported similar behavior in polycrystalline nickel^35,41^. Additionally, the thermal component of the short-elastic interaction τ* represented only 10% of the short-range interactions (effective stress τ_eff_ = τ* + τ_µ_ with τ_µ_ the athermal component of the stress) on the strain range investigated, which allowed us to then consider the athermal component of the effective stress measurements and the contribution of other internal stresses.

### Internal stress

**Figure 3:**
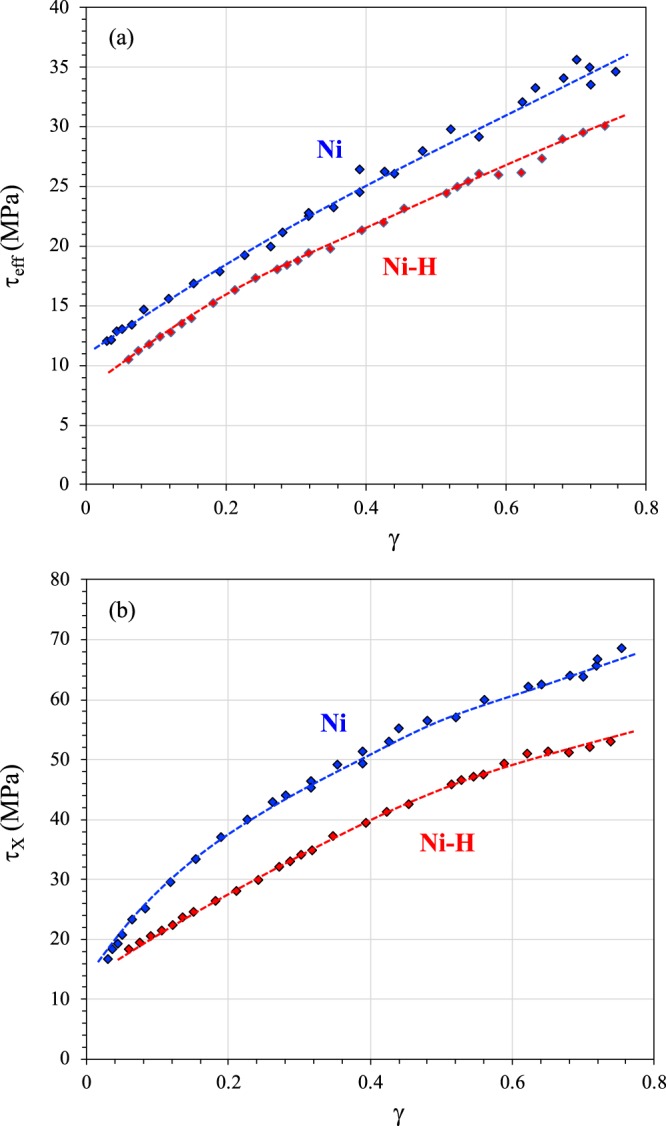
Effective stress (**a**) and back stress (**b**) as a function of shear strain for Ni and Ni-H.

The effective τ_eff_ and back stress τ_X_ investigated by Dickson’s partition applied to each unloading sequence is shown in Fig. 3. Both stresses can be associated with the internal stress that developed with the formation of a dislocation pattern. According to the composite model^27,42,43^, effective stress represents the stress required locally for a dislocation to move (short-range interactions) in cells (“soft phase”). In contrast, back stress is linked to the local straining process (plastic strain incompatibilities between the “hard phase”, the dislocation walls, and the “soft phase”, the cell) that introduces long-range interactions with mobile dislocations in the cell^42,44^. Hydrogen content affects both components, with a decrease in these stresses with the addition of the solute. Expressed differently, these results show a softening effect on the athermal components of stress (τ_X_ and τ_eff_ ≈τ_µ_ in accordance with the fact that τ* represents only 10% of the short-range interactions). The hydrogen softening process observed on the short-range interactions with mobile dislocations (τ_eff_) could be related to the shielding mechanism in which hydrogen reduces elastic pair interactions between two dislocations^6–11^.

Recent investigations into the impact of hydrogen on elastic properties (E, μ the Young modulus and shear modulus, respectively) demonstrated that point defects produced by the incorporation of hydrogen had a higher impact on the elastic constant than the solute^6–11^. The decrease in elastic properties with the addition of hydrogen has been well documented recently in the superabundant vacancies (SAV) mechanism^11^. Vacancy formation and clustering induce a long-range internal strain which reduces apparent elastic stiffness coefficients to a greater extent than hydrogen self-stress. Consequently, the softening behavior observed for short-range interactions in dislocations can be directly related to the point defects and clusters of vacancies produced during the initial incorporation of hydrogen, as confirmed by TEM observations^11,45^ and our present measurement (around 3.8 10^−4^ V/Ni for the unstrained sample after a hydrogen pre-charging to 7 wppm compared with 2.8 10^−24^ V/Ni at P_H2_ = 1 bar and 300 K for pure nickel single crystals^46^). This interpretation is mainly based on the hypothesis that vacancy concentration does not change during strengthening, which is not the case, as previously shown^47,48^. In the present work, C_v_ was measured for a plastic strain of 0.7 for a sample with (around 10^−4^ V/Ni) or without hydrogen (around 3.10^−5^ V/Ni). These first results show that pre-charging sample had a vacancy concentration one order higher than a pure strained nickel sample without hydrogen. Additionally, the initial SAV introduced with pre-charging hydrogen (around 3.8 10^−4^ V/Ni for the unstrained sample after hydrogen pre-charging to 7 wppm) seemed to be decreased by the strengthening process (around 10^−4^ V/Ni), which suggests that vacancies and vacancy clusters are partially annihilated by dislocations. Consequently, the process which consists in a reduction of apparent elastic stiffness coefficients by the vacancies seems to be more effective for samples with an initial incorporation of hydrogen in the unstrained sample than a strained sample with and without pre-charging sequence.

### Dislocation patterns

**Figure 4:**
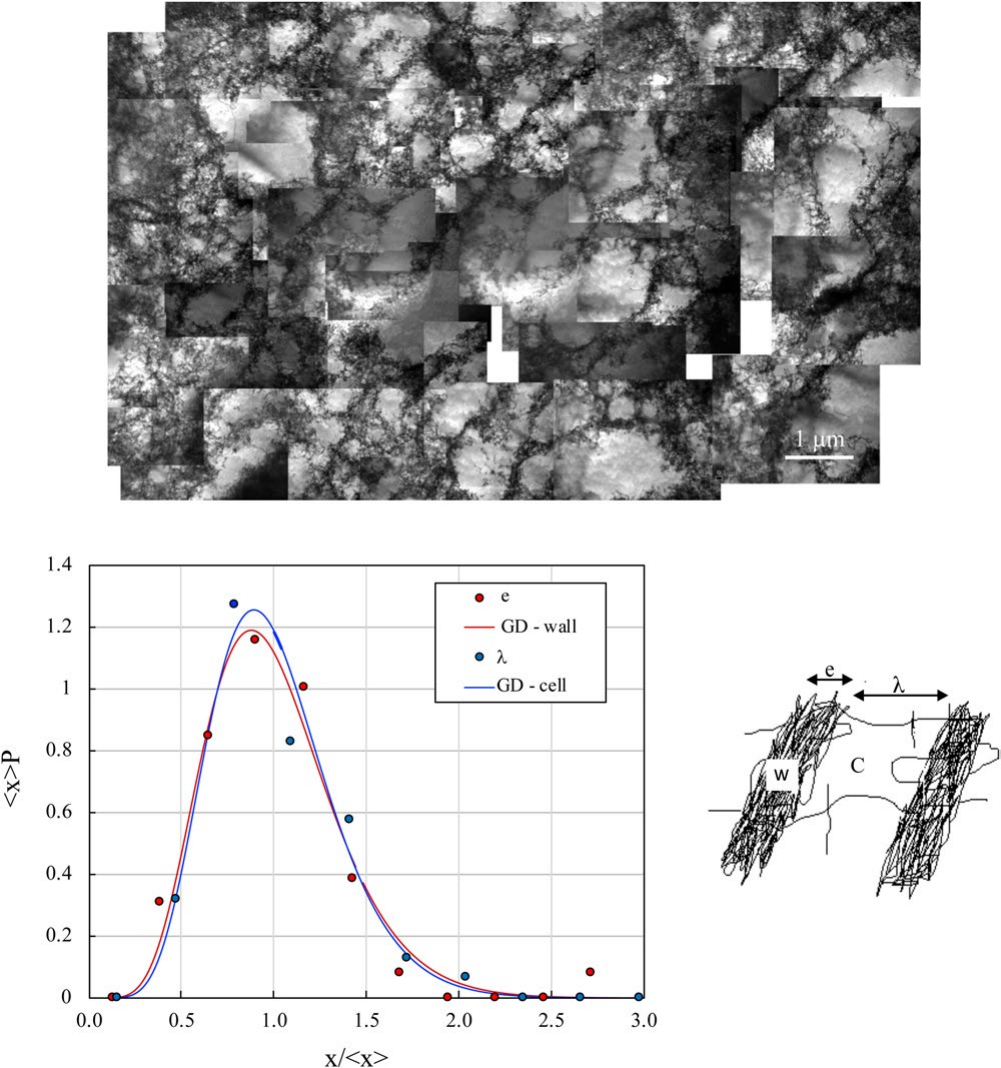
TEM illustration of the organization of dislocations for a plastic strain of 0.7 with a hydrogen pre-charging sample at 7 ± 0.5 wppm (**a**). Normalized e and λ distributions with *x* equals e or λ (**b**). The probability density function normalized by the average dimension <x> P *versus* x/<x> has an asymmetric morphology. This scaled distribution corresponds to a gamma distribution (GD): $f(X)=\frac{1}{\Gamma .{\beta }^{\alpha +1}}{X}^{\alpha }.exp[-\frac{X}{\beta }]$ where X = x/<x>, σ^2^ is the variance with β = σ^2^ = 1/(1 + α) and Γ is defined as an Euler integral^26,27^.

To improve our understanding of the effect of solute on back stress τ_X_ softening, we analyzed the organization of dislocation by TEM for a plastic strain of 0.7 with a hydrogen pre-charging sample at 7 ± 0.5 wppm (Fig. 4). We observed equiaxed cells (type I pattern, Fig. 4a), which is a common feature for a single-crystal strengthened with tensile loading near <001>^27^. As previously reported, a fixed area was scanned and statistical analyses were performed to obtain average wall thickness (*e*), cell size (*λ*) and density of dislocation in the wall and cell phases, ρ_w_ and ρ_c_ respectively (Fig. 4c). The normalized distribution of <*x*> P *versus x*/<*x*> is presented for *x* equals *e* or *λ* (P is the probability of obtaining a size *x*) and can be described by a gamma distribution with a variance σ^2^, as shown in Fig. 4b (details in references^22,26,27^). For Ni-H, the variance was 0.11 for *e* and 0.12 for *λ*. These values are not so different and correspond to the value obtained in our previous work with strained nickel without hydrogen and oriented for multiple-slips (001)^27^. As was extensively discussed previously, this variance corresponds to the formation of Incidental Dislocation Boundaries (IDBs) in a quasi-random dislocation trapping phenomenon. Consequently, the addition of hydrogen to this tensile orientation did not modify the formation process of type I dislocation cells (equiaxed cells with incidental dislocations trapped in walls). In contrast, the average wall thickness <*e*> and cell size (*λ*) were affected by the solute content (<*e*> (Ni) = 227 nm and <*e*> (Ni-H) = 193 nm, and in the other part <*λ*> (Ni) = 875 nm and <*λ*> (Ni-H) = 639 nm). Both decreased with hydrogen content, as previously reported by Girardin *et al*.^22^ and Wang *et al*.^23^, but the effect was less pronounced than for Geometrical Necessary Boundary (GNBs) spacing^22^ (see Figure 8a of reference^22^). The average density of dislocation decreased on addition of hydrogen (<ρ_c_> (Ni) = 4.8 10^13^ m^−2^ and <ρ_c_> (Ni-H) = 3.6 10^13^ m^−2^, and in the other part <ρ_w_> (Ni) = 9. 10^14^ m^−2^ and <ρ_w_> (Ni-H) = 5.2 10^14^ m^−2^). The impact was more significant for the dislocation density in walls than in cells. According to the composite model initially proposed by Mughrabi and developed by various authors^42,43^, we can deduce the stress improved by dislocation in walls *τ*_*w*_ as:

1 $${\tau }_{w}=\frac{\tau -(1-{f}_{w})\cdot {\tau }_{eff}}{{f}_{w}}$$

where *f*_w_ = *e*/(*e* + *λ*) is the “hard phase” fraction (wall phase) and the hypothesis of the stress applied is expressed as $\tau ={f}_{w}{\tau }_{w}+(1-{f}_{w}){\tau }_{c}$ and that *τ*_*c*_ = *τ*_*eff*_. Using the average value obtained for each parameter *τ*, *τ*_*eff*_, and f_W_ at a plastic strain of 0.7, Eq. (1) gave a value of τ_w_ for Ni and a Ni-H of 397 and 257 MPa, respectively. As was observed in terms of dislocation density in the walls ρ_w_, hydrogen also induced a softening in terms of local stress in this phase τ_w_. To measure the consequence of these microstructural features on long-range internal stress (LRI), we used the composite model^42,43^. Based on this model, long-range internal stress can be expressed as^42,44^:

2 $${\tau }_{X}(LRI)={f}_{w}({\tau }_{w}-{\tau }_{c})=\alpha \mu b{f}_{w}(\sqrt{{\rho }_{w}}-\sqrt{{\rho }_{c}})$$

with α an average value of a coefficient which describes the elastic interaction between slips, b is the Burgers vector and *f*_w_ = *e*/(*e* + *λ*) the “hard phase” fraction (wall phase), which was 20.5%. and 23.2% for Ni and Ni-H, respectively. According to Eq. (2), the decrease in long-range internal stress is mainly associated with a decrease in dislocation density in the walls through a shielding effect, leading to a reduction in elastic interactions between dislocations and consequently promoting a reduction in dislocation trapping frequency. As a consequence, despite the fact that hydrogen reduced the length scales (*e*, *λ*) associated with the dislocation pattern and increased the fraction of wall f_W_, the decrease in hardening rate θ_ΙΙ_ was attributed mainly to a reduction in long-range internal stress induced by IDBs. This result is different from that reported for the GNBs that developed during tensile strained nickel single-crystals oriented for single-slips^22^. In this orientation, the formation of GNBs defined the mean free path of mobile dislocations as *λ*. The biggest impact of hydrogen in this situation was a large decrease in GNB spacing (*λ*) and consequently an increase in strain hardening in stage II. As a result, hydrogen content had a different impact on GNBs and IDBs in relation to the processes which initiate their formation. For GNBs, the length path *λ* between walls is the main factor affected by hydrogen, whereas for IDBs, the dislocation density in walls is largely dependent on hydrogen solute.

## Discussion

The hydrogen softening observed on stress τ, activation volume V/b^3^ and hardening rate θ_ΙΙ_ can be discussed in term of the elastic shielding process with the classic “textbook of plasticity” (elementary processes of dislocation theory) introduced by Delafosse^2^. According to this approach, the shielding effect, based on a degradation of the apparent elastic properties, is characterized by a screening index S(a_i_) = a_i_(H)/a_i_(0) with a_i_(H) and a_i_(0) a physical and/or mechanical parameter a_i_, (see below) with and without hydrogen, respectively.

By extending the linear-elastic theory of the equilibrium between interstitial solid solution atoms and host metal lattices used in several studies^6–10^, we have recently determined the degradation of elastic properties as a function of hydrogen alone, monovacancies and vacancy cluster concentrations using an analytical approach verified by DFT calculations^11^. This degradation is due more to vacancy clusters than hydrogen itself^11^. According to recent experimental data^11,49^, the screening index for elastic properties (E, Young modulus) S(E) = E(H)/E(0) decreases linearly with hydrogen content to a value around 0.82 when hydrogen solubility is reached. Despite the fact that the reduction of apparent elastic stiffness coefficients by the vacancy clusters is more effective for samples with an initial incorporation of hydrogen than after a pre-strained one, the vacancy concentration obtained at a strain of 0.7 (C_V_ = 10^−4^ V/Ni) gave a screening index of S(E) = E(H)/E(0) = 0.95^11^. The screening index impacts all the elementary plasticity processes.

**Figure 5:**
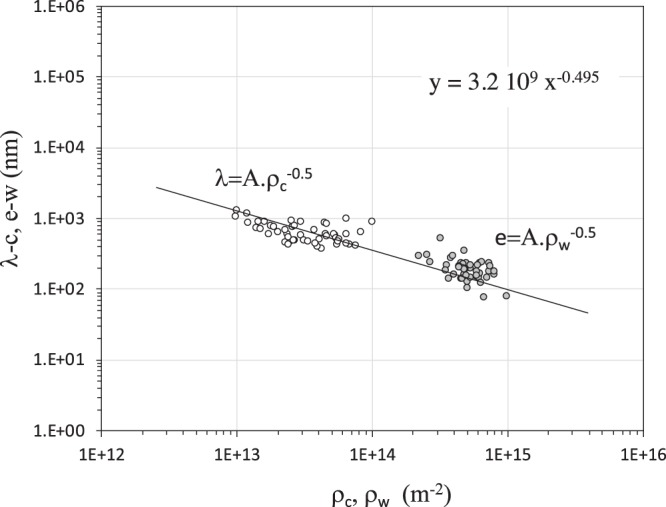
Correlation between thickness e and spacing λ and the density of dislocations in walls ρ_w_ and in inter-wall spacing ρ_c_ (Ni-H, γ = 0.7), respectively.

Recently, an extensive collection of experimental data was gathered for nickel to improve the well-known self-organization process of dislocation resulting from a monotonic deformation of (001) nickel single crystals^27^. Length scales and scaling laws for dislocation cells developed during tensile loading were established. It was shown that the parameters λ and e were inversely proportional to the square root of the dislocation density ρ_c_ and ρ_w_, respectively, with the same slope A (λ = Α*ρ*_*c*_^−1/2^ and e = Α*ρ*_*w*_^−1/2^) of 3.9 in multiple slip conditions for a (001) nickel single crystal orientation and polycrystalline microstructure^26,27^. In the present work, with the addition of hydrogen, from 100 measurements we obtained a similitude law with a slope A of 3.2 (Fig. 5). This value is lower than pure nickel straining in the same conditions with a shielding parameter of S(A) = 0.82. By combining the strengthening relation based on the composite model and similitude relations defined previously, shear stress is obtained^27^:

3 $$\tau =(2\alpha \mu Ab){\lambda }^{-1}$$

Based on the knowledge of shielding coefficients associated with τ, μ, λ and A we can evaluate the impact of hydrogen on the strengthening coefficient α using Eq. (3): S(α) = S(τ) × S(λ)/[S(A) × S(μ)]. For a hydrogen content of 7 wppm and a shear strain of 0.7, a screening factor S(α) of 0.75 was obtained with S(τ) = 0.8, S(λ) = 0.73, S(A) = 0.82 and S(μ) = 0.95 (according to the recent data of Hachet *et al*.^11^). As shown, S(α) < 1, indicating that hydrogen lowered the strengthening coefficient α which defines the elastic interaction between dislocations.

The relationship between length scale λ (cell size) and hardening rate θ is the final similar relation we can develop. It is based on two sets of equations: one describing the change in dislocation density as a function of strain and the second being the Taylor relationship. If annihilation processes are not taken into account, it was demonstrated previously^27^ that hardening rate is then given by:

4 $$\theta =(\frac{\alpha \mu k}{2\sqrt{{\rho }_{c}}}){\lambda }^{-1}=\frac{\alpha \mu k}{2A}$$

with k a constant which depends on the number of slip systems operating during strengthening and expresses the capacity to produce dislocations during strengthening. Consequently, based on Eq. (4), the shielding coefficients associated with k can be expressed as S(k) = S(θ) × S(A)/[S(α)) × S(μ)] and gave 0.74, with a hardening rate θ_II_ in stage II of 194 MPa for Ni and 124 MPa for Ni-H (S(θ) = 0.64), S(A) = 0.82, S(α) = 0.75 and S(μ) = 0.95. Consequently, hydrogen directly impacted the kinetics of dislocation multiplication (k).

The last impact of hydrogen on elementary processes of plasticity can be questioned on the basis of the change in activation volume. According to Fig. 2, the B slopes obtained for Ni-H and Ni on the V/b^3^ *versus* τ* diagram are the same (Fig. 2b). Using the expression of B as a function of α, μ and k_0_, we can deduce S(k_0_) = 1/(S(μ) × S(α)). The “screening index” associated with the mean free path of dislocations k_0_/$\sqrt{\rho }$. S(k_0_) = 1.57 was higher than 1, which illustrates that the addition of hydrogen increased the mean free path of dislocations.

To conclude, hydrogen solute promotes a shielding process which induces softening behavior in both phases (walls and cells) for multiple slip conditions of tensile strengthening of (001) nickel single crystals. The amplitude of this effect is characterized by a screening index S(a_i_) with a value of around 0.75 to 0.85, which impacts the kinetics of dislocation multiplication (k) and the elastic properties and strengthening coefficient (μ, α), respectively. Additionally, an increase in the mean free path of dislocation was obtained.

The prediction of the amplitude of softening by the shielding model initially introduced by Sofronis *et al*.^6^ is in progress, but at this stage it is important to say that our recent work^11,21^ shows that the degradation of Young’s modulus with the introduction of hydrogen is mainly due to the vacancy cluster formation (SAV) process. The present results suggest that clusters of superabundant vacancies (SAV) are probably the main defect, which explains the softening at different microstructural scales: forest hardening (short-range interactions) and dislocation patterning (long-range interactions). In this work, the measurement of vacancy density showed that the density introduced by the hydrogen pre-charging step is important (3.8 10^−4^ V/Ni) but was partially restored during strengthening. Thus, the question of the change in the initial vacancy concentration during strengthening is important and needs further investigation.

## Conclusion

To summarize, the impact of the addition of hydrogen on the tensile behavior of nickel single-crystals oriented to promote multiple-slips was different from that of the same crystal oriented for a single-slip. Stress softening was related to a decrease in short and long-range internal stress. This is discussed in terms of a modification in the dislocation pattern and the shielding effect which occurs in both phases (wall and cell). The effect was formalized in a textbook of mechanisms of plasticity by a screening factor S equal to 0.8 ± 0.05. Additionally, it was suggested that clusters of superabundant vacancies (SAV) were probably the main defects, which explains the hydrogen-induced softening process. This last point needs to be supported by additional measurements.

## Methods

### Sample preparation

The material investigated was high purity (100) single-crystal Ni (99.999%) in the form of a cylindrical bar provided by Goodfellow. Tensile specimens oriented along the <100> crystallographic direction were machined by STEEC using the electro-erosion method. Mechanical and electro-chemical polishing was performed as part of the procedure we used in our previous work^36,50^. An estimation of the dislocation density of the as-received material gave an average value of less than 10^−10^ m^2^.

### Charging hydrogen conditions

The electrochemistry charging method was used to obtain a hydrogen content of 7 ± 0.5 wppm in 0.1 M NaOH at 298 K under galvanostatic polarization (−10 mA/cm^2^) for 72h^51^. Solutions were continuously deaerated by argon gas at 1.4 bar.

Hydrogen content was measured using Thermal Desorption Spectroscopy (TDS). These analyses were performed with a Jobin Yvon Horiba EMGA-621W hydrogen analyzer composed of an impulsion furnace system coupled with a thermal conductivity detector. Initial or after-straining conditions, vacancy concentration induced by hydrogen incorporation (SuperAbundant Vacancies SAV^46,52^) and/or tensile strain were measured using Differential Scanning Calorimetry (DSC)^11,45,51^. After the introduction into the Q100 TA Instruments DSC test bench, the sample was heated from room temperature up to 673 K at increments of 10 K/min. The change in heat flow as a function of the temperature highlighted the presence of an exothermic peak associated with the annihilation of vacancies. Based on the fact that the area of this peak corresponds to the stored energy of the vacancies, the concentration of vacancies was deduced^53^.

### Mechanical tests

Three kinds of mechanical tests were performed using the typical fatigue micro-machine (Kammrath & Weiss, 5 kN) at a strain rate of 10^−3^ s^−1^. A standard tensile test, a multi-step stress relaxation test and a sequence of loading-unloading steps were carried out to determine strain hardening, the change in apparent activation volume and internal stress for different levels of plastic strain. Strain hardening under tension of fcc alloys is traditionally characterized using the Kocks and Mecking representation τ.θ *versus* τ where θ = dτ/dγ_p_ is the hardening rate^33^. The stress relaxation test is one of the common methods used to study time-dependent ductility. Since the total strain remains constant during the relaxation test, the plastic strain rate can be deduced as follows: $d{\gamma }_{p}/dt=-\,\frac{1}{E}d\tau /dt$. Consequently, the apparent activation volume for each stress relaxation step up to the stabilization of stress was: ${V}_{a}={k}_{B}T[\frac{\partial Ln({\dot{\gamma }}_{p})}{\partial \tau }]$ with ${\dot{\gamma }}_{p}$ the plastic strain rate, and the thermal component of effective stress τ* was evaluated. Back stress τ_X_ and effective stress τ_eff_ were determined from the unloading sequence feature on the τ *versus* γ curve, as previously illustrated^42,44^. This stress partition was performed after unloading at different plastic strain levels. During the unloading sequence, a linear regime (the elastic domain) was observed until a reverse yield stress τ_r_ was reached. Then the partition could be expressed as follows:

5 $${\tau }_{eff}=\frac{\tau -{\tau }_{r}}{2}+\frac{{\tau }^{\ast }}{2}\,{\rm{and}}\,{\tau }_{X}=\tau -{\tau }_{eff}$$

These components offer the opportunity to question the contribution of long-range internal stress improved by mobile dislocations τ_X_, and short-range τ_eff_ interactions required locally for a dislocation to move, respectively. The latter internal stress is typically decomposed in terms of τ_µ_ the athermal component and τ* the thermal component, as follows: τ_eff_ = τ* + τ_µ_.

### TEM statistical analysis of some topological parameters

The organization of dislocation was investigated using a JEOL JEM 2011 electron microscope operating at 200 kV. Foils for transmission electron microscopy (TEM) were thinned in a double twin-jet electro-polisher using electrolytes under the conditions described previously^26,27,42^. The mapping zone was generally equivalent to 100 × 200 mm^2^ and about 50 images were collected from the same part of the TEM foil. A statistical analysis of dislocation density and distribution (size of cells and wall thickness) was conducted on 50 to 100 measurements for each plastic strain studied. Dislocation densities were measured using the standard intersection method^27,42^ from TEM micrographs collected by Digital Micrograph™ software. The possible errors and the imprecision in measurements of the dislocation densities by TEM combined with the line intersection method have recently been discussed^27^ and support the present analyses.

### Impact statement

Hydrogen-induced softening of internal stress was first clearly established in tensile strengthening nickel single crystal oriented for multiple-slips. This effect is mainly associated with the shielding of elastic interactions in relation to the SAV process.
